# Plasma soluble cellular prion protein reflects ischemic stroke severity and is associated with circulating CD4^+^ T cell immune responses

**DOI:** 10.3389/fimmu.2026.1801975

**Published:** 2026-04-22

**Authors:** Shuqin Hou, Yue Lang, Xiang Yin, Weiguanliu Zhang, Xiaona Li, Li Cui

**Affiliations:** Department of Neurology, The First Hospital of Jilin University, Changchun, China

**Keywords:** biomarker, CD4+ T cells, cellular prion protein, ischemic stroke, T-helper subsets

## Abstract

**Background:**

The soluble form of cellular prion protein (PrP^C^) in plasma has been investigated as a biomarker in multiple conditions, yet its relationship with ischemic stroke severity remains unclear. Soluble PrP^C^ is also implicated in immune-cell activation and inflammatory regulation. Here, we examined whether plasma soluble PrP^C^ is associated with stroke severity and circulating CD4^+^ T cell immune responses in patients with acute ischemic stroke.

**Methods:**

In this single-center prospective cohort study, we consecutively enrolled patients with acute ischemic stroke admitted to the Department of Neurology, The First Hospital of Jilin University (Changchun, China) between June 2023 and October 2024 within 48 h of symptom onset (for wake-up stroke, onset was defined as the last known well). Fasting venous blood was collected the morning after admission. Stroke severity was categorized by admission NIHSS as mild (≤6) or moderate-to-severe (>6), and volunteers from the Health Examination Center served as controls. Circulating CD4^+^ T cells and Th1/Th2/Th17 subsets were quantified by flow cytometry, and soluble PrP^C^ concentrations in plasma were measured by ELISA.

**Results:**

Plasma soluble PrP^C^ concentrations were higher in patients with ischemic stroke than in controls (1.16 [0.74, 1.99] ng/mL vs 0.54 [0.36, 0.65] ng/mL; P < 0.0001) and were further increased in the moderate-to-severe subgroup (1.91 [1.10, 3.18] ng/mL vs 0.86 [0.58, 1.40] ng/mL; P < 0.01). Plasma soluble PrP^C^ was independently associated with admission NIHSS (B = 2.085, 95% CI 1.176-2.994; P < 0.001). Among stroke patients, plasma soluble PrP^C^ was positively associated with Th2 proportion (ρ = 0.73; P < 0.001) and negatively associated with the Th1/Th2 ratio (ρ = -0.66; P < 0.001); both associations remained significant after covariate adjustment (Th2: B = 1.504, 95% CI 1.166–1.841; Th1/Th2: B = −2.374, 95% CI −3.427–−1.322; both P < 0.001).

**Conclusions:**

Plasma soluble PrP^C^ is elevated early after ischemic stroke and is associated with stroke severity and a Th2-skewed circulating CD4^+^ T-cell profile. These findings support plasma soluble PrP^C^ as a candidate acute-phase marker linked to post-stroke immune dysregulation.

## 1 Introduction

Ischemic stroke triggers a systemic immune response. Disruption of the blood-brain barrier allows brain-derived antigens and inflammatory mediators to enter the circulation, activating peripheral immune cells and promoting their recruitment into the ischemic brain (1). T lymphocytes infiltrate the central nervous system after stroke in a temporally ordered manner and contribute to both tissue injury and repair (2, 3). CD4^+^ T cells differentiate into effector subsets, including Th1, Th17, and Th2 cells, which shape post-stroke inflammation through distinct cytokine programs: Th1/Th17 responses are generally pro-inflammatory, whereas Th2 responses are considered relatively anti-inflammatory (3). Accordingly, characterizing early peripheral CD4^+^ T-cell responses may provide mechanistic insight and potential therapeutic targets in ischemic stroke.

Cellular prion protein (PrP^C^), encoded by the prion protein gene (PRNP), is a cell-surface glycoprotein broadly expressed across tissues and enriched in neurons and astrocytes (4, 5). Beyond its membrane-anchored form, PrP^C^ undergoes proteolytic processing and can be released as soluble fragments into extracellular fluids, either associated with extracellular vesicles or as a free soluble protein (6). Circulating soluble PrP^C^ has been proposed as a biomarker in several neurologic conditions. For example, plasma soluble PrP^C^ levels are increased after intracerebral hemorrhage and aneurysmal subarachnoid hemorrhage and are associated with disease severity and 90-day outcomes (7, 8). PrP^C^ has also been implicated as a biomarker in non-neurological diseases; in chronic kidney disease, urinary PrP^C^ is elevated and correlates with renal function indices, consistent with renal epithelial secretion under endoplasmic reticulum stress (9). However, the behavior and clinical relevance of plasma soluble PrP^C^ after ischemic stroke have not been well defined.

Experimental studies further indicate that PrP^C^ is upregulated in hypoxic regions and peri-infarct tissue after cerebral ischemia, with increased PRNP mRNA expression, suggesting a role in endogenous responses to ischemic injury (10–14). PrP^C^ has been linked to multiple signaling pathways (e.g., cAMP/PKA, MAPK, PI3K/Akt, and Src-family kinases) and to processes relevant to recovery, including modulation of glutamatergic signaling, angiogenesis, and neurogenesis (15, 16).

In addition to its neurobiological roles, soluble PrP^C^ has been implicated in immune regulation. Soluble PrP^C^ can modulate monocyte/macrophage functions and natural killer-cell activation through pathways including ERK and NF-κB signaling (17–19). In adaptive immunity, PrP^C^ expression increases upon T-cell activation, and genetic or experimental perturbation of PrP^C^ can alter cytokine production by T-helper subsets (20, 21). These observations raise the possibility that circulating soluble PrP^C^ may be linked to peripheral immune phenotypes after ischemic stroke.

In our previous animal study, splenic CD4^+^ PrP^C+^ T lymphocytes increased after middle cerebral artery occlusion and reperfusion (MCAO/R). PRNP deletion promoted Th1/Th17 polarization with enhanced pro-inflammatory cytokine production and aggravated neuronal apoptosis, whereas PRNP overexpression favored a Th2-dominant phenotype and was associated with neuroprotection (22).

Based on these findings, we aimed to evaluate whether plasma soluble PrP^C^ is associated with stroke severity and peripheral CD4^+^ T-cell immune responses in acute ischemic stroke. We therefore quantified plasma soluble PrP^C^ and circulating CD4^+^ T-cell subset distributions in patients with ischemic stroke and evaluated their relationships with clinical severity and short-term outcomes.

## 2 Materials and methods

### 2.1 Study population

We consecutively enrolled 55 patients with anterior-circulation ischemic stroke (age 18–80 years) admitted to the Department of Neurology, The First Hospital of Jilin University, within 48 h of symptom onset. For wake-up strokes, onset was defined as the last known well. All patients underwent non-contrast CT and MRI diffusion-weighted imaging (DWI) for hemorrhage exclusion and infarct confirmation; vascular imaging (CTA or MRA) was obtained when available. Anterior-circulation stroke was defined by infarct location within the internal carotid artery-middle cerebral artery (ICA-MCA) and/or anterior cerebral artery (ACA) territories on DWI (supported by CTA/MRA when available). During the same period, 24 volunteers undergoing routine physical examinations at the Health Examination Center were recruited as controls. Stroke severity was categorized by admission NIHSS as mild (≤6) or moderate-to-severe (>6). Demographic and clinical data were collected, including age, sex, admission NIHSS, vascular risk factors, and comorbidities. No formal sample size calculation was performed because this was an exploratory study; the sample size was determined by feasibility and consecutive recruitment during the prespecified period.

The exclusion criteria were as follows:

1. patients who received intravenous thrombolysis or endovascular intervention;
2. patients with infectious diseases, autoimmune diseases, or those receiving immunosuppressive or immunomodulatory therapy;
3. patients with a history of cerebral infarction or intracerebral hemorrhage within the past year;
4. patients with severe hepatic or renal dysfunction, defined as alanine aminotransferase (ALT) or aspartate aminotransferase (AST) levels exceeding three times the upper limit of normal, serum creatinine levels >265 μmol/L, heart failure, or malignant tumors.

Functional outcomes were assessed at 3 months by trained medical personnel via telephone interviews using the modified Rankin Scale (mRS). Favorable outcomes were defined as no symptoms to mild disability (mRS 0–2), whereas unfavorable outcomes were defined as moderate disability to death (mRS 3–6).

This study was approved by the Clinical Research Management Committee of The First Hospital of Jilin University, and written informed consent was obtained from all participants or their legal guardians prior to enrollment.

### 2.2 Flow cytometry

On the morning of the first day after admission, 3 mL of fasting peripheral venous blood was collected into sodium heparin anticoagulant tubes. PBMCs were isolated by density-gradient centrifugation and incubated in RPMI 1640 supplemented with PMA (25 ng/mL) and ionomycin (1 μM) at 37 °C with 5% CO_2_ for 5 h. GolgiStop™ Protein Transport Inhibitor was added to inhibit cytokine secretion. The collected cells were fixed and permeabilized using Cytofix™ Fixation Buffer, followed by incubation with a cocktail of fluorochrome-conjugated antibodies in the dark. Intracellular cytokine staining was performed using a BD Human Th1/Th2/Th17 phenotyping kit (BD Pharmingen™, San Diego, CA, USA; Cat. #560751), which includes CD4 (PerCP-Cy5.5), IFN-γ (FITC), IL-4 (APC), and IL-17A (PE). Compensation was calculated using single-stained compensation beads, and gates were defined using isotype controls. Data acquisition was performed using a BD FACS Canto II flow cytometer, and data analysis was conducted with FlowJo software. The immune cell subsets analyzed in this study were defined as follows: Th1 (CD4^+^IFN-γ^+^), Th2 (CD4^+^IL-4^+^), and Th17 (CD4^+^IL-17A^+^).

### 2.3 Enzyme-linked immunosorbent assay

Blood samples were centrifuged at 4 °C (4000 rpm, 10 min) to obtain plasma. Plasma soluble PrP^C^ concentrations were quantified according to the manufacturer’s instructions using a commercial Human Prion Protein (PrP^C^) ELISA kit (JL13080; Jianglai Biotechnology, China).

### 2.4 Statistical analysis

Statistical analyses were performed using SPSS 26.0 (IBM, Armonk, NY, USA), and figures were generated with GraphPad Prism 9.5 (GraphPad Software, San Diego, CA, USA). Data were assessed for normality and homogeneity of variance and are presented as mean ± SD or median (IQR), as appropriate. Analyses involving T-cell subsets were performed using available cases. Group comparisons used Student’s t-test/one-way ANOVA or Mann-Whitney U/Kruskal-Wallis tests with Dunn’s *post hoc* multiple-comparisons correction when applicable; categorical variables were compared using χ² or Fisher’s exact tests. Associations were evaluated using Spearman’s rank correlation. Linear regression was used to assess the relationship between plasma soluble PrP^C^ and admission NIHSS with multicollinearity assessment and residual diagnostics; soluble PrP^C^ was analyzed on its original scale (no log-transformation). Predictors of 3-month outcome were evaluated using binary logistic regression with outcome dichotomized as favorable (mRS ≤ 2) vs unfavorable (mRS > 2), after checking collinearity. All tests were two-sided, and P < 0.05 was considered statistically significant.

## 3 Results

### 3.1 Baseline characteristics of the study population

Initially, 395 patients with acute ischemic stroke were screened for eligibility among which 336 patients were excluded according to prespecified criteria and 4 patients were lost to follow-up (**Supplementary Figure 1**). In the final analysis, a total of 55 patients with ischemic stroke were enrolled in this study, including 31 patients with mild stroke and 24 patients with moderate-to-severe stroke, along with 24 non-stroke controls. The clinical characteristics of the study population are summarized in **Table 1**. Overall, baseline characteristics were broadly comparable among the three groups. However, the prevalence of hypertension (P = 0.02) and the proportion of individuals with a history of smoking (P = 0.035) were significantly higher in the stroke groups than in the non-stroke control group. Plasma soluble PrP^C^ was successfully measured in all patients (n = 55). Flow cytometry data were unavailable for 4 patients due to accidental sample loss during processing; therefore, analyses involving T-cell subsets were performed in 51 patients.

**Table 1:** Baseline characteristics of the study population.

Variable	Mild stroke (n = 31)	Moderate-to-severe stroke (n = 24)	Controls (n = 24)	P value
Age, years (mean ± SD)	59.13 ± 11.11	63.54 ± 8.38	58.63 ± 7.36	0.130
Male sex, n (%)	24 (77.4)	16 (66.7)	17 (70.8)	0.668
Pre-stroke mRS, median (IQR)	0 (0–0)	0 (0–0)	0 (0–0)	—
Hypertension, n (%)	21 (67.7)	16 (66.7)	8 (33.3)	0.020
Diabetes mellitus, n (%)	10 (32.3)	3 (12.5)	6 (25.0)	0.234
Coronary artery disease, n (%)	5 (16.1)	3 (12.5)	1 (4.2)	0.457
Atrial fibrillation, n (%)	1 (3.2)	3 (12.5)	0 (0)	0.183
Hyperlipidemia, n (%)	20 (64.5)	12 (52.2)	11 (45.8)	0.364
Hyperhomocysteinemia, n (%)	10 (32.3)	2 (8.7)	4 (16.7)	0.106
Smoking history, n (%)	18 (58.1)	13 (54.2)	6 (25.0)	0.035
Alcohol consumption, n (%)	17 (54.8)	13 (54.2)	10 (41.7)	0.574

Data are presented as mean ± standard deviation (SD), median (interquartile range, IQR), or number (percentage), as appropriate. P values were calculated using one-way analysis of variance (ANOVA) or the Kruskal-Wallis test for continuous variables, and the χ² test or Fisher’s exact test for categorical variables, as appropriate.

### 3.2 Association between plasma soluble PrP^C^ and disease severity after ischemic stroke

**Figure 1:**
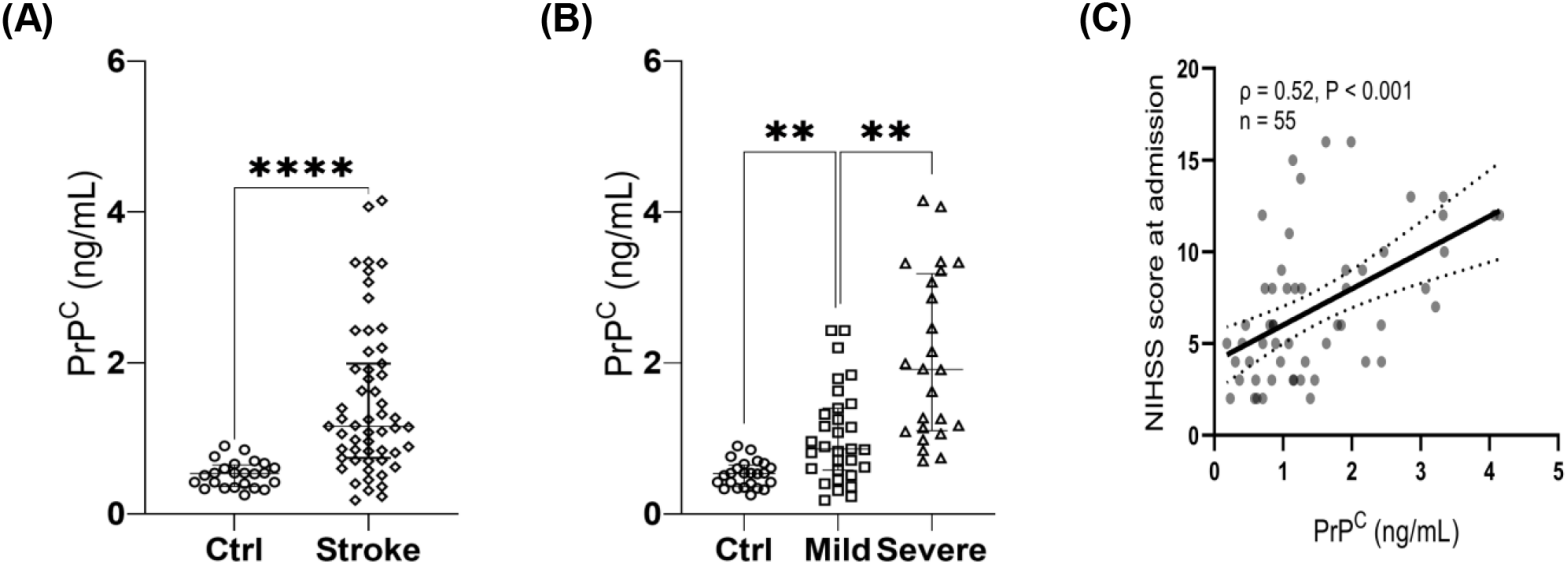
Association between plasma soluble PrP^C^ concentration and disease severity after ischemic stroke. **(A)** Plasma soluble PrP^C^ concentrations in non-stroke controls (Ctrl, n = 24) and patients with ischemic stroke (Stroke, n = 55). **(B)** Plasma soluble PrP^C^ concentrations across Ctrl (n = 24) and ischemic stroke severity subgroups (Mild, n = 31; Severe, n = 24). For brevity, the moderate-to-severe subgroup is labeled as “severe” in the figure. **(C)** Spearman correlation was performed in stroke patients only (controls excluded; n = 55). Data are presented as median (IQR); each symbol represents one participant. Group comparisons were performed using nonparametric tests (two-group comparison: Mann–Whitney U test; multiple-group comparison: Kruskal-Wallis test followed by Dunn’s *post hoc* test, as applicable). PrP^C^ values represent plasma soluble PrP^C^ levels quantified by ELISA and are expressed in ng/mL. NIHSS, National Institutes of Health Stroke Scale; PrP^C^, cellular prion protein. **P < 0.01; ****P < 0.0001.

Plasma soluble PrP^C^ concentrations were higher in patients with ischemic stroke than in non-stroke controls (1.16 [0.74, 1.99] ng/mL vs 0.54 [0.36, 0.65] ng/mL; P < 0.0001) (**Figure 1A**). Patients with moderate-to-severe stroke also had higher plasma soluble PrP^C^ levels than those with mild stroke (1.91 [1.10, 3.18] ng/mL vs 0.86 [0.58, 1.40] ng/mL; P < 0.01) (**Figure 1B**).

Among stroke patients, plasma soluble PrP^C^ correlated positively with admission NIHSS (Spearman’s ρ = 0.52; P < 0.001) (**Figure 1C**). In multivariable linear regression, plasma soluble PrP^C^ remained independently associated with NIHSS after adjustment for age, sex, and vascular risk factors (B = 2.085, 95% CI 1.176-2.994; P < 0.001).

### 3.3 Changes in circulating CD4^+^ T-cell proportions after ischemic stroke

**Figure 2:**
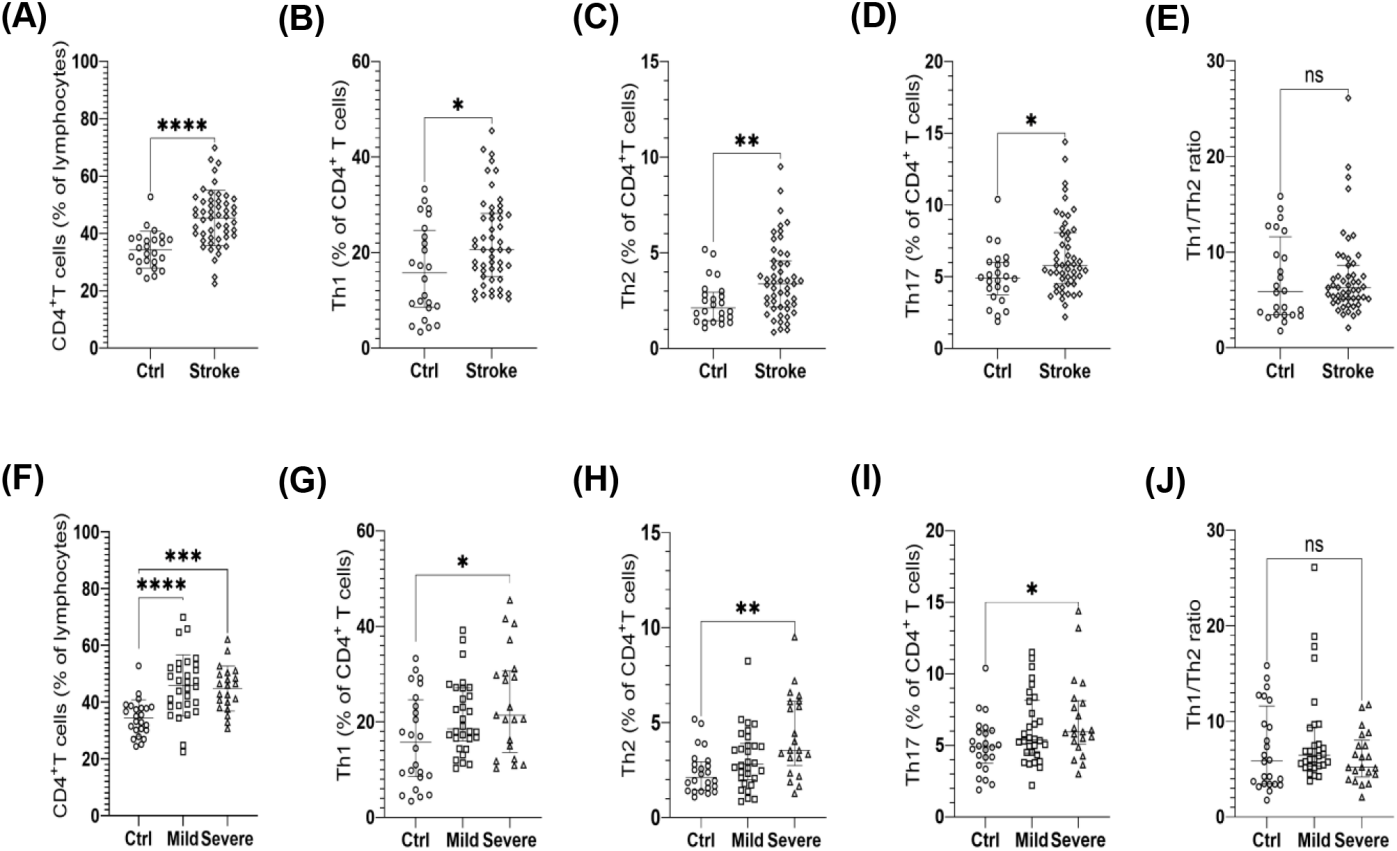
Circulating CD4⁺ T-cell proportions and helper T-cell subset profiles in ischemic stroke. Immune profiles are shown for no-stroke controls (Ctrl) and ischemic stroke patients (Stroke) **(A–E)**, and across NIHSS-based severity subgroups (Ctrl, Mild, Moderate-to-severe) **(F–J)**; for brevity, the moderate-to-severe subgroup is labeled as “severe” in the figure. Panels depict **(A, F)** CD4⁺ T cells as a percentage of lymphocytes, **(B, G)** Th1 cells as a percentage of CD4⁺ T cells, **(C, H)** Th2 cells as a percentage of CD4⁺ T cells, **(D, I)** Th17 cells as a percentage of CD4⁺ T cells, and **(E, J)** the Th1/Th2 ratio. Each symbol represents one participant. For panels **A** and **F**, data are presented as mean ± SD and comparisons were performed using an unpaired two-tailed t-test **(A)** or one-way ANOVA **(F)**, as appropriate. For panels **B–E** and **G–J**, data are presented as the median with interquartile range (IQR) and comparisons were performed using the Mann–Whitney U test **(B–E)** or Kruskal–Wallis test **(G–J)**. For three-group panels **(F–J)**, brackets indicate pairwise comparisons between Ctrl and Severe (not the overall three-group test), with *post hoc* multiple-comparisons correction applied where appropriate. Sample size: Ctrl, n = 24; Stroke, n = 55. T-cell subset data were unavailable for 4 stroke patients (Mild, 1 missing; Severe, 3 missing); therefore, analyses involving Th subsets and the Th1/Th2 ratio used n = 51 stroke patients (Mild, n = 30; Severe, n = 21). *P < 0.05, **P < 0.01, ***P < 0.001, ****P < 0.0001; ns, not significant.

Compared with non-stroke controls, patients with ischemic stroke exhibited higher proportions of CD4^+^ T cells (45.42 ± 9.65% vs 34.36 ± 6.47%, P < 0.0001), Th1 cells (20.60 [15.00–28.20]% vs 15.75 [8.52–24.65]%, P < 0.05), Th2 cells (3.38 [2.17–4.58]% vs 2.12 [1.45–2.94]%, P < 0.01), and Th17 cells (5.79 [4.51–8.08]% vs 4.91 [3.75–6.01]%, P < 0.05) (**Figures 2A–E**).

In the moderate-to-severe stroke subgroup, the proportions of CD4^+^ T cells (44.80 ± 7.93% vs 34.36 ± 6.47%, P < 0.001), Th1 cells (21.40 [13.60–30.65]% vs 15.75 [8.52–24.65]%, P < 0.05), Th2 cells (3.54 [2.74–6.12]% vs 2.12 [1.45–2.94]%, P < 0.01), and Th17 cells (5.94 [5.08–8.12]% vs 4.91 [3.75–6.01]%, P < 0.05) were higher than those in controls. No significant differences were observed between the mild and moderate-to-severe stroke subgroups (all P > 0.05) (**Figures 2F–J**). No significant correlations were observed between circulating Th-cell subset proportions and admission NIHSS (all P > 0.05). The Th1/Th2 and Th17/Th2 ratios did not differ significantly between patients with ischemic stroke and controls or across stroke severity subgroups. (**Supplementary Figure 2**).

### 3.4 Association between plasma soluble PrP^C^ and CD4^+^ T-cell subset proportions in ischemic stroke

**Figure 3:**
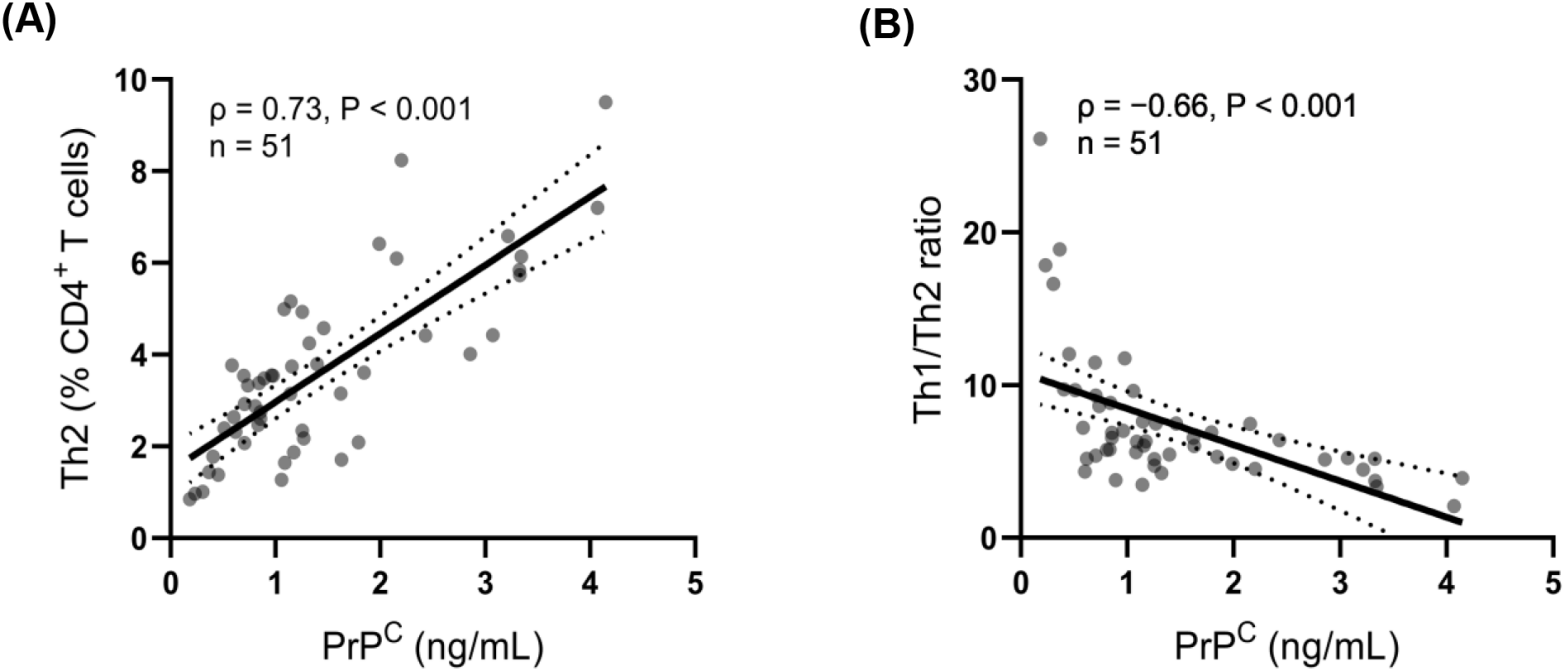
Plasma soluble PrP^C^ concentration correlates with Th2 proportion and the Th1/Th2 ratio in patients with ischemic stroke. Scatter plots show the associations between plasma soluble PrP^C^ (x-axis; quantified by ELISA, ng/mL) and **(A)** Th2 cells (% of CD4^+^ T cells) and **(B)** the Th1/Th2 ratio in stroke patients with available paired data (n = 51). Associations were assessed using Spearman’s rank correlation (A, ρ = 0.73, P < 0.001; B, ρ = −0.66, P < 0.001). Solid lines indicate a simple linear fit for visualization only, and dashed lines indicate 95% confidence intervals.

Spearman’s correlation analysis in patients with ischemic stroke showed that soluble PrP^C^ concentration was positively correlated with the proportion of Th2 cells among CD4^+^ T cells (Spearman’s ρ = 0.73, P < 0.001) (**Figure 3A**) and negatively correlated with the Th1/Th2 ratio (Spearman’s ρ = −0.66, P < 0.001) (**Figure 3B**).

Consistently, in multivariable linear regression models adjusting for age, sex, and relevant vascular risk factors, soluble PrP^C^ concentration remained independently associated with a higher proportion of Th2 cells among CD4^+^ T cells (B = 1.504, 95% CI 1.166–1.841; P < 0.001) and a lower Th1/Th2 ratio (B = −2.374, 95% CI −3.427 to −1.322; P < 0.001). No comparable associations were observed in the non-stroke control group.

### 3.5 Plasma soluble PrP^C^ and short-term outcome after ischemic stroke

A total of 55 patients with ischemic stroke were included and stratified into a favorable outcome group (3-month mRS ≤ 2, n = 32) and an unfavorable outcome group (3-month mRS > 2, n = 23). Baseline clinical characteristics and peripheral blood parameters are summarized in **Table 2**. Admission NIHSS score and soluble PrP^C^ concentration were higher in patients with unfavorable outcomes.

**Table 2:** Baseline characteristics of ischemic stroke patients stratified by 3-month functional outcome (mRS).

Variable	Favorable outcome (mRS ≤ 2, n = 32)	Unfavorable outcome (mRS > 2, n = 23)	P value
Age, years	59.20 ± 11.56	64.24 ± 7.48	0.140
Male sex, n (%)	25 (78.1)	15 (65.2)	0.289
Hypertension, n (%)	23 (71.9)	14 (60.9)	0.391
Diabetes mellitus, n (%)	10 (31.3)	3 (13.0)	0.117
Hyperlipidemia, n (%)	19 (59.4)	13 (59.1)	0.983
Hyperhomocysteinemia, n (%)	9 (28.1)	3 (13.6)	0.320
Coronary heart disease, n (%)	5 (15.6)	3 (13.0)	1.000
Atrial fibrillation, n (%)	1 (3.1)	3 (13.0)	0.298
Smoking, n (%)	18 (56.3)	13 (56.5)	0.984
Alcohol consumption, n (%)	18 (56.3)	12 (52.2)	0.765
Admission NIHSS score	4.00 [3.00–5.75]	10.00 [8.00–13.00]	<0.001
3-month mRS score	1.00 [0.00–2.00]	4.00 [3.00–5.00]	—
PrP^C^, ng/mL	0.93 [0.60–1.41]	1.25 [0.91–3.20]	0.012
CD4^+^ T cells, %	45.87 ± 10.67	44.77 ± 8.18	0.694
Th1, %	19.85 [15.75–27.20]	20.60 [13.60–30.65]	0.572
Th2, %	2.90 [2.09–4.29]	3.54 [2.55–5.97]	0.193
Th17, %	5.46 [4.51–8.45]	5.94 [4.56–7.79]	0.954
Th1/Th2 ratio	6.21 [5.26–8.03]	6.28 [4.31–8.72]	0.284

Data are presented as mean ± SD, standard deviation, median (IQR, interquartile range), or number (percentage), as appropriate. P values were calculated using the unpaired Student’s t test or Mann–Whitney U test for continuous variables, and the χ² test or Fisher’s exact test for categorical variables, as appropriate. PrP^C^ values represent plasma soluble PrP^C^ levels quantified by ELISA and are expressed in ng/mL. CD4^+^ T cells are expressed as a percentage of lymphocytes. Th1/Th2/Th17 are expressed as percentages of CD4^+^ T cells. NIHSS, National Institutes of Health Stroke Scale; mRS, modified Rankin Scale; PrP^C^, cellular prion protein.

Given that mRS is an ordinal scale, Spearman correlation was used. Three-month mRS scores were positively correlated with age (ρ = 0.27, P < 0.05, n = 55), admission NIHSS score (ρ = 0.92, P < 0.001, n = 55), and soluble PrP^C^ concentration (ρ = 0.37, P < 0.01, n = 55). Binary logistic regression analyses were performed to identify predictors of unfavorable outcome. In univariable models, admission NIHSS score (OR = 4.250, 95% CI 1.799–10.239, P = 0.001) and soluble PrP^C^ concentration (OR = 2.418, 95% CI 1.256–4.655, P < 0.01) were significant predictors of unfavorable outcome. Given that age is a well-established prognostic factor, age, admission NIHSS score, and plasma soluble PrP^C^ concentration were entered into the multivariable model; after adjustment, only admission NIHSS score remained independently associated with unfavorable outcome (OR = 6.973, 95% CI 1.813–26.816, P < 0.01) (**Table 3**).

**Table 3:** Univariable and multivariable binary logistic regression analyses for unfavorable 3-month outcome.

Variable	Univariable		Multivariable	
	OR (95%CI)	P	OR (95%CI)	P
Age (years)	1.056 (0.994–1.121)	0.079	1.083 (0.914–1.283)	0.355
Admission NIHSS score	4.250 (1.799–10.239)	0.001	6.973 (1.813–26.816)	0.005
PrP^C^ (ng/mL)	2.418 (1.256–4.655)	0.008	0.312 (0.059–1.654)	0.171

Unfavorable outcome was defined as a 3-month mRS score > 2. ORs were estimated using binary logistic regression. The multivariable model included age, admission NIHSS score, and PrP^C^ concentration. PrP^C^ values represent plasma soluble PrP^C^ levels quantified by ELISA and are expressed in ng/mL. NIHSS, National Institutes of Health Stroke Scale; mRS, modified Rankin Scale; PrP^C^, cellular prion protein.

## 4 Discussion

In this study, plasma soluble PrP^C^ was elevated early after anterior-circulation ischemic stroke and was associated with neurological severity. Prior work has suggested that PrP^C^ may serve as a candidate biomarker in several neurologic and systemic conditions (7–9, 23–25). In experimental stroke models, PrP^C^ expression increases in ischemic brain regions and has been reported to correlate with injury severity (14). Consistent with these observations, we found higher plasma soluble PrP^C^ levels in stroke patients than in controls, further elevations in moderate-to-severe stroke, and an independent association with admission NIHSS. These findings support that circulating soluble PrP^C^ may reflect the extent of acute ischemic injury and may serve as a severity-linked biomarker in the early phase of stroke.

Post-stroke immunity is dynamic and can contribute to both secondary injury and repair. Acute ischemia and blood-brain barrier disruption promote peripheral immune activation, trafficking, and cytokine release, whereas counter-regulatory anti-inflammatory programs limit excessive inflammation (26). We observed increased proportions of total CD4^+^ T cells and Th1, Th2, and Th17 subsets after stroke, suggesting broad activation of peripheral helper T-cell responses during the acute stage. Severity-related differences in these proportions were directionally consistent but did not reach statistical significance, and no significant correlations were found between circulating helper T-cell subset proportions and admission NIHSS. These findings may reflect the temporal complexity of post-stroke immune responses, as well as the limited sample size of the present study. Importantly, because blood samples were obtained at only one early time point, longitudinal sampling in larger cohorts will be important to define the temporal trajectory of circulating T-helper responses after ischemic stroke.

Notably, plasma soluble PrP^C^ was associated with a Th2-skewed phenotype among stroke patients, showing a positive correlation with Th2 proportion and a negative correlation with the Th1/Th2 ratio. Although the present study was observational and cannot establish causality or define the underlying mechanism, several biologically plausible explanations may be considered. First, previous studies suggest that PrP^C^ may participate in immune-cell differentiation and lineage regulation. PrP^C^ has been reported to be expressed in long-term hematopoietic stem cells (27) and to remain expressed during lymphoid and monocytic differentiation, whereas its expression is downregulated during granulocytic differentiation (28), supporting a potential role in hematopoietic and immune-cell fate regulation. Second, soluble PrP^C^ has been shown to modulate monocyte/macrophage function and natural killer-cell activation through signaling pathways including ERK and NF-κB (17–19), raising the possibility that it may also influence helper T-cell polarization through signaling-dependent mechanisms, either directly or indirectly through changes in the cytokine milieu. Third, previous studies have shown that PrP^C^ can co-precipitate with the T-cell receptor complex (29), and that PrP^C^ silencing enhances T-cell activation, TCR signaling, and differentiation toward pro-inflammatory Th1 and Th17 phenotypes (30). Consistent with these observations, our previous experimental work in cerebral ischemia/reperfusion models showed that PRNP deletion promoted Th1/Th17 polarization, whereas PRNP overexpression favored a Th2-dominant phenotype (22). Together, these findings provide a plausible mechanistic framework linking PrP^C^ to helper T-cell polarization. At the same time, indirect mechanisms should also be considered. Because PrP^C^ has been implicated in the regulation of multiple immune-cell populations, its association with Th-cell balance after stroke may be mediated, at least in part, through effects on monocytes/macrophages, antigen-presenting cells, or other immune cells that shape T-helper differentiation. Importantly, however, the present clinical study measured circulating soluble PrP^C^ rather than cell-surface PrP^C^, and the major source of soluble PrP^C^ after stroke remains unclear. Therefore, we cannot determine whether soluble PrP^C^ directly contributes to Th-cell polarization or instead reflects broader injury-associated immune dysregulation. In addition, post-stroke immunity is highly dynamic: excessive early inflammation may aggravate injury, whereas later immune responses may contribute to debris clearance and tissue repair. Further longitudinal and mechanistic studies across different stages of stroke are needed to clarify the context-dependent role of PrP^C^ in post-stroke immune regulation.

Although higher plasma soluble PrP^C^ levels were observed in patients with unfavorable 3-month outcomes and were significant in univariable analysis, only admission NIHSS remained an independent predictor in the logistic model. This suggests that the prognostic information carried by soluble PrP^C^ may largely overlap with initial neurological severity rather than provide independent prognostic value. Accordingly, soluble PrP^C^ may currently be better interpreted as a severity-linked biomarker than as an independent predictor of functional outcome. Future studies with larger cohorts, prespecified prognostic models, and external validation will be required to determine whether soluble PrP^C^ provides incremental prognostic value beyond established clinical measures and biomarkers.

Several limitations of this study should be acknowledged. First, this was a single-center study with a modest sample size, which may have limited statistical power and generalizability. Second, soluble PrP^C^ and circulating T-cell subsets were measured at a single early time point only. Given the dynamic nature of post-stroke immune responses, we were therefore unable to assess their temporal evolution. Third, the observational design precludes mechanistic inference and causal interpretation. In particular, we could not determine whether soluble PrP^C^ directly influences helper T-cell polarization or instead reflects tissue injury and post-stroke immune dysregulation. Fourth, the cellular source of circulating soluble PrP^C^ after stroke was not examined, and no experimental validation was included in this clinical study. Further multicenter studies with larger sample sizes, longitudinal sampling, integrated biomarker analyses, and mechanistic validation are needed to clarify the biological and clinical relevance of soluble PrP^C^ in ischemic stroke.

## 5 Conclusion

In this single-center cohort of anterior-circulation acute ischemic stroke, plasma soluble PrP^C^ was elevated early after stroke and was independently associated with admission NIHSS. Higher soluble PrP^C^ was associated with a Th2-skewed CD4^+^ T-cell profile at an early time point, supporting soluble PrP^C^ as a candidate acute-phase marker linked to post-stroke immune dysregulation.

This study was limited by its single-center design, modest sample size, and single time-point sampling; mechanistic inferences cannot be made. Larger multicenter studies with longitudinal sampling and experimental validation are warranted.

## Data Availability

The original contributions presented in the study are included in the article/**Supplementary Material**. Further inquiries can be directed to the corresponding author.
