# Supplementary material for: Plasma soluble cellular prion protein reflects ischemic stroke severity and is associated with circulating CD4+ T cell immune responses
Source: Front Immunol. 2026 Apr 22;17:1801975. doi: 10.3389/fimmu.2026.1801975 (PMC13143613; doi:10.3389/fimmu.2026.1801975)
Supplement: Supplementary file 2 [file DataSheet2.docx]

Supplementary Material

# Supplementary Figures and Tables

## Supplementary Figures

**Supplementary Figure 1**


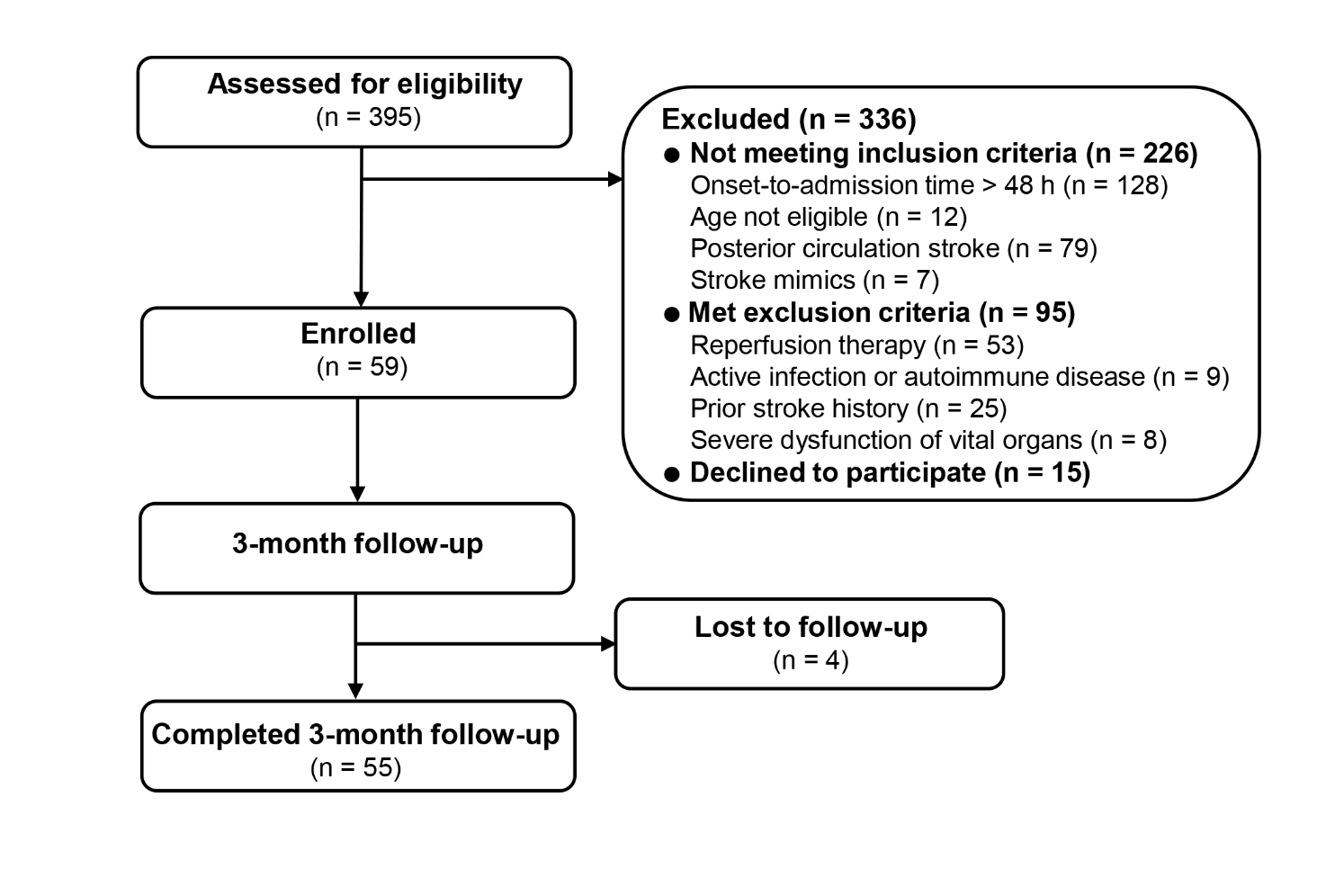


**Supplementary Figure 1.** A total of 395 patients were screened for eligibility. Of these, 336 were excluded due to not meeting inclusion criteria (n = 226), meeting exclusion criteria (n = 95), or declining participation (n = 15). Common reasons for exclusion included onset-to-admission time > 48 h, posterior circulation stroke, receipt of reperfusion therapy, previous stroke history, and severe dysfunction of vital organs. Ultimately, 59 patients were enrolled in the study, and 55 completed the 3-month follow-up, while 4 patients were lost to follow-up.

**Supplementary Figure 2**


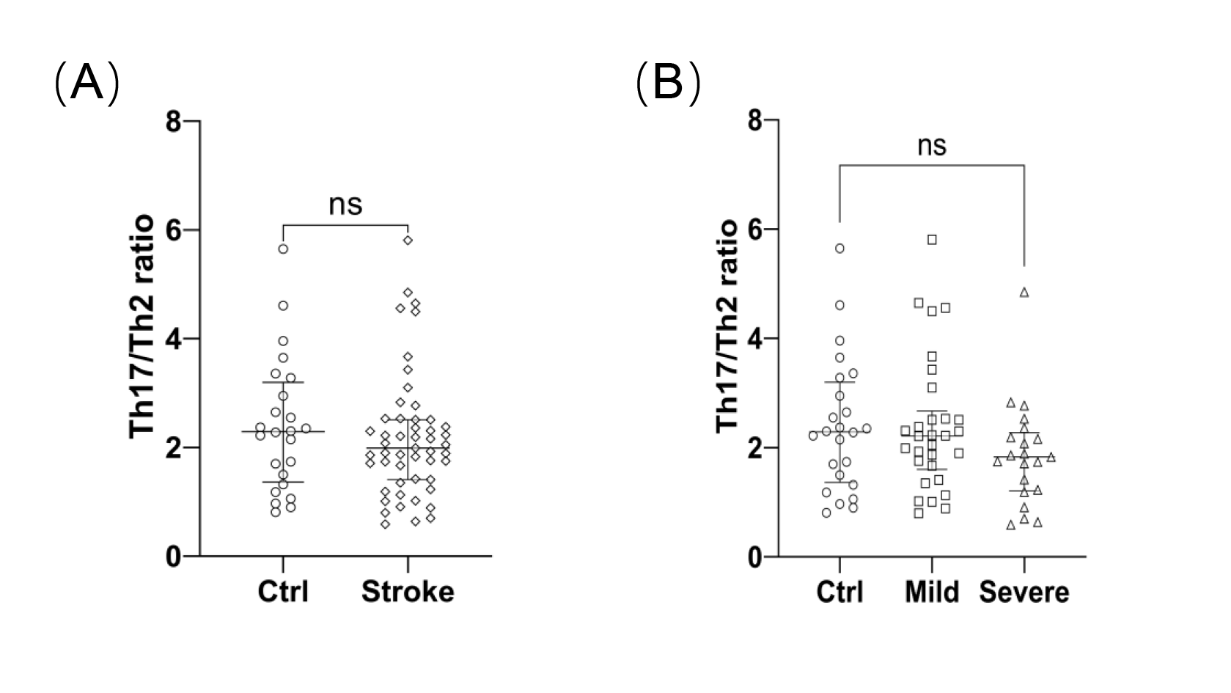


**Supplementary Figure 2**. Th17/Th2 ratio in patients with ischemic stroke and controls

The Th17/Th2 ratio was compared between non-stroke controls (Ctrl) and patients with ischemic stroke (Stroke), as well as across stroke severity subgroups (Ctrl, Mild, Severe). For brevity, the moderate-to-severe subgroup is labeled as “severe” in the figure. Each dot represents one participant. Data are presented as median with interquartile range (IQR). Group comparisons were performed using the Mann–Whitney U test (two-group comparison) or the Kruskal–Wallis test with post hoc multiple-comparisons correction (three-group comparison), as appropriate. No significant differences were observed between groups. ns, not significant.
